# Menopause and suicide: A systematic review

**DOI:** 10.1177/17455057251360517

**Published:** 2025-10-09

**Authors:** Olivia Hendriks, Jason C. McIntyre, Abigail K. Rose, Laura Sambrook, Daniel Reisel, Clair Crockett, Louise Newson, Pooja Saini

**Affiliations:** 1 School of Psychology, Liverpool John Moores University, UK; 2 University College London, UK; 3 Newson Health Menopause and Wellbeing Centre, Stratford-Upon-Avon, UK

**Keywords:** menopause, suicidality, hormones, primary ovarian insufficiency, cultural and social factors

## Abstract

**Background:**

The menopausal transition is a critical phase in a woman’s life marked by hormonal fluctuations that can result in a wide variety of physical and psychological symptoms. These symptoms vary in strength and their negative impacts on women’s health and well-being. One of the most severe impacts of (peri)menopause is increased vulnerability to suicidality in some women, yet no systematic review has examined the holistic relationship regarding this potential link.

**Objectives:**

To examine the relationship between the menopausal transition and suicidality, and identify menopause-related factors contributing to increased suicide risk.

**Design:**

A systematic review was conducted in accordance with PRISMA guidelines.

**Data Sources:**

MedLine, CINAHL, PsychINFO, Web of Science and Cochrane Library were searched for studies addressing both menopause and suicidality. Studies were screened independently by two reviewers. Data extraction focused on suicidal ideation, attempts, and completed suicide among menopausal women. The quality of included studies was assessed using the Mixed Methods Appraisal Tool.

**Results:**

Nineteen studies published between 1987 and 2025 met the inclusion criteria. Of the 19 studies, 16 (84%) reported an association between the menopausal transition and increased suicidality, with 7 studies specifically noting this association in perimenopausal women. Hormonal changes, pre-existing mental health conditions, physical symptoms, and limited social support emerged as key factors associated with increased suicide risk. Three studies did not find a significant link.

**Conclusion:**

There is some evidence of an association between the menopausal transition and suicidality, particularly during perimenopause, though conclusions are limited by study design and heterogeneity. The review highlights the importance of integrating mental health support within menopause care and suggests further research to clarify the mechanisms underpinning suicide risk during the menopausal transition. Enhanced screening and supportive interventions may benefit menopausal women experiencing suicidality.

## Introduction

The menopausal transition is a significant, mutable phase in a woman’s life which typically occurs between the ages of 45 and 55 and marks the termination of menstrual activity, covering shifts from pre-, peri- and post-menopause.^1^ This transition is characterised by fluctuating levels of steroid hormones such as oestrogen, testosterone and progesterone, which may lead to various physiological and psychological changes of differing severity.^2–4^ Common physiological symptoms can include hot flashes and night sweats, which can affect up to 75% of menopausal women and may persist for several years.^4^ These can sometimes cause further, more acute vasomotor symptoms such as full-body chills and heart palpitations, substantially disrupting routine activities including sleep.^5^ Additionally, oestrogen loss can reduce bone density, increasing the risk of osteoporosis and bone fractures.^6^ Disruptions such as these can contribute to irritability, fatigue and overall daily discomfort, impacting quality of life, which may exacerbate existing mental health concerns or lead to the development of new ones.^5^

The hormonal fluctuations that occur during the menopausal transition can lead to a range of cognitive and psychological symptoms such as memory lapses, difficulty concentrating, mood swings, anxiety, low mood and reduced libido.^7–9^ Systematic reviews and meta-analyses exploring mental health issues during menopause have found that women are more vulnerable to both anxiety and depression.^10–14^ This is particularly evident in perimenopause (the few years leading up to the cessation of menstrual periods when vasomotor symptoms begin and changes in the menstrual cycle occur^15^ and, less severely, in early postmenopause (the few years after menopause) where hormonal fluctuations are more pronounced.^5,16^ These fluctuations, specifically the decline in oestrogen and progesterone, can have a significant impact on brain chemistry as they both play important roles in the regulation of neurotransmitters such as serotonin and norepinephrine, which are necessary for mood stabilisation.^17^ Studies have shown that decreased oestrogen levels during the menopausal transition are associated with a higher prevalence of anxiety,^14^ depressive symptoms and major depressive episodes, particularly in women with a history of depression.^5,18^ Additionally, oestrogen replacement therapy can be effective in alleviating depression and anxiety symptoms during menopause, further supporting the posited role of oestrogen in mood regulation.^19^

Overall, research suggests that the interaction between menopausal physiological symptoms and psychological distress can cause a decline in mental well-being. A 2021 survey revealed that 86% of women aged 46–60 reported experiencing mental health issues during menopause; 58% reported low energy and lack of motivation, 53% suffered from low mood and depression, 50% experienced anxiety, 42% reported anger and mood swings, and 33% expressed feelings of worthlessness.^20^ Despite this clear impact of the menopausal transition on mental ill-health for a significant number of women, suicidality during menopause has received relatively little attention. Yet, suicide is generally a major public health concern. According to the WHO^21^, more than 700,000 people die by suicide each year, with women showing different patterns and predictors compared to men. In general, women are three times more likely to attempt suicide than men,^22^ however, suicide research traditionally focuses on the mortality of suicidal behaviours, which typically favours men, who die from suicide two to four times more than women.^23^ Compared to men, women face unique risk factors for suicide including a higher prevalence of psychosocial issues (e.g. domestic violence, physical, emotional, and sexual abuse, lower levels of education, single marital status, and financial instability).^24,25^ Women also tend to be more vulnerable to these stressors and experience a higher incidence of mental health disorders.^26^

In 2023, the age-specific female suicide rate in the United Kingdom was its highest since 1994 and most severe among women aged 45–64.^27^ Similar findings have been reported in the USA and Australia.^28,29^ These age ranges coincide with stages of menopause, and some have suggested that the biological changes and negative symptomatology associated with this transition may contribute to increased suicide risk.^30^ However, this relationship is likely to be complex,^3^ with multiple potential exacerbating factors, such as treatment delays and absence of social support,^31,32^ contributing to an increased risk of suicidal ideation and suicidal behaviours.^33^

Globally, there are calls for more women-specific health research, and there is a growing recognition that the menopausal transition can increase the risk of significant mental and physical health harms.^34^ However, there is no synthesised evidence regarding the potential association between menopause and suicidality while accounting for the complex interplay of biological, psychosocial, cultural, and intersectional factors. A previous systematic review by Martin-Key et al.^35^ synthesised 28 studies and offered important insights into the relationship between menopause and suicidality. However, it did not fully explore how cultural and ethnic contexts, early menopause (including primary ovarian insufficiency [POI]), and comorbid physical health conditions influence this relationship. Therefore, this systematic review aims to build on and extend that work by addressing these gaps. Specifically, it synthesises existing research on menopause and suicidality, with a particular focus on early menopause, cultural influences, comorbidities, and implications for integrated care approaches in clinical practice.

## Method

### Protocol

The protocol was registered with International Prospective Register of Systematic Reviews (PROSPERO) ID: CRD42023475768. Available from: crd.york.ac.uk/PROSPERO/display_record.php?RecordID=475768.

### Search strategy

**Figure 1.**
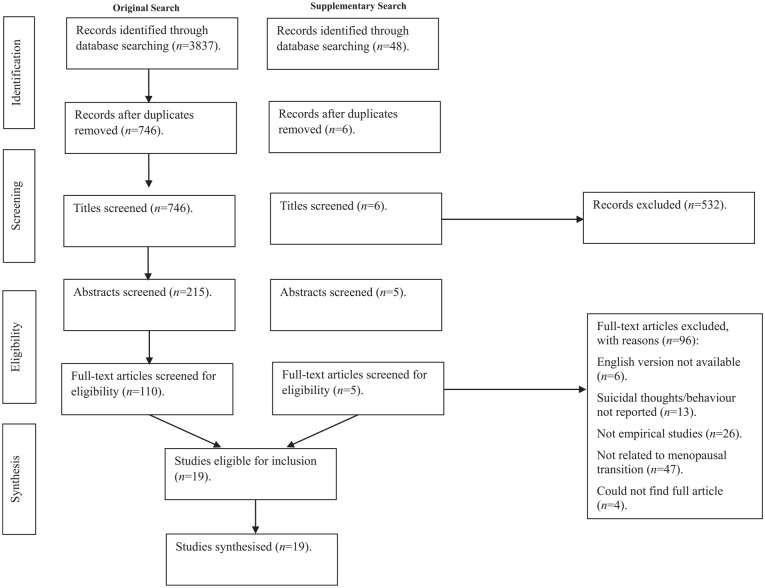
PRISMA flow diagram showing the flow of studies within the review.

The reporting of this systematic review conforms to the PRISMA 2020 statement^36^ and checklist (see Figure 1). Electronic databases were searched from inception to 21.11.23 (with updated searches conducted in January 2024 and April 2025): (MedLine, CINAHL, PsycINFO, Web of Science and Cochrane Library) for two key concepts: (1) suicide and self-harm and (2) menopause. Formal electronic searches were supplemented by a manual search of reference sections in eligible papers and review articles. Search terms involved a combination of free text and indexing terms (e.g. MeSH).

### Eligibility criteria

Studies were included if they reported data on people experiencing the menopausal transition, with a clearly described measure of suicide, suicidality or self-harm. Suicidality encompassed suicidal ideation, suicide attempts and completed suicide, while self-harm was defined as any intentional self-injury, regardless of suicidal intent. Outcome variables were identified using relevant literature and included suicidal ideation, suicide attempts, completed suicide and associated psychological and physical health outcomes. The specific eligibility criteria for the included studies are outlined in Table 1.

**Table 1.** Inclusion and exclusion criteria.

Inclusion criteria Population(s) and condition of interest	Population(s): actively menopausal women (i.e. perimenopausal and still experiencing menstrual periods). Condition of interest: suicidal ideation, self-harm, suicide attempt.
Intervention(s)/exposure	Actively menopausal women experiencing suicidal behaviours and/or thoughts, and/or non-suicidal self-injury.
Comparators	None.
Outcome	Factors influencing suicidality in actively menopausal women, such as Hormone Replacement Therapy (HRT; i.e. oestrogen, testosterone, progesterone), mental health support, and lifestyle factors.
Setting	None.
Study designs	Qualitative, mixed methods, randomised controlled trials, and non-randomised quantitative studies.
Exclusion criteria	Non-English language studies where translation could not be obtained. Studies reporting solely on general mental health without a mention of suicidality. Studies focused solely outside of the active menopausal period (e.g. premenopausal or postmenopausal women).

### Study screening and selection

Two authors (OH and LS) independently reviewed titles, abstracts and full texts against the eligibility criteria. Discrepancies were resolved through discussion. There was high agreement between authors on inclusion/exclusion decisions (85%), with a kappa value of 0.77, indicating substantial agreement between raters.^37^

### Data extraction and quality assessment

Eligible full texts were subjected to data extraction and quality assessment by the primary author. For data extraction, we systematically gathered information on suicidality outcomes (including suicidal ideation, suicide attempts, and suicide deaths), menopausal stage (pre-, peri- or post-menopause) and participant characteristics (e.g. age, ethnicity). Additional data extracted included physical health comorbidities (e.g. body mass index (BMI) and metabolic status), study design, setting and funding sources when available. When data were unclear or not explicitly stated in the original study reports, we did not make assumptions and recorded these as not reported.

The Mixed Methods Appraisal Tool (MMAT)^38^ was used to assess methodological quality of included studies.^39,40^ All studies found in the review were included in data synthesis, regardless of risk of bias/quality assessment. Studies were scored based on how rigorously they applied appropriate methods within their respective design categories. While cross-sectional studies have inherent limitations, such as the inability to infer causality, the MMAT permits these to be rated as high quality when they demonstrate methodological rigour in sampling, measurement and analysis.^39,41^ This approach is supported by broader evidence that well-conducted cross-sectional studies can be assessed as methodologically sound within their scope.^42,43^

### Data synthesis

Narrative synthesis using the framework developed by Popay et al.^44^ was conducted. This is a method for synthesising findings from multiple studies in a systematic review, particularly when quantitative meta-analysis is not feasible due to the heterogeneity in study designs, populations, or outcomes. It provides a structured synthesis of the findings, using descriptions and themes to summarise patterns, relationships and variations across studies. Given this heterogeneity, a segmented synthesis approach was used conceptually: while all studies were included in a unified summary table for clarity, findings from quantitative, qualitative and mixed-methods studies were interpreted within their respective methodological contexts to maintain conceptual clarity and avoid conflating statistical associations with thematic insights. This approach allowed for meaningful interpretation across diverse forms of evidence. Given the complexity of the topic, narrative synthesis was particularly well suited to exploring how menopausal symptoms, mental health, and suicidality intersect, while also incorporating the contextual depth offered by qualitative findings. The relationships within and across studies were explored by examining the similarities and differences between them (see Supplemental Table 1). This approach has been used successfully in similar mental health and suicidality reviews where diverse study designs were included.^45–47^ This process followed a transparent and systematic review framework consistent with PRISMA and established guidance for narrative synthesis,^44^ ensuring a comprehensive and rigorous integration of the included evidence.

## Results

The search yielded 3,837 records from which 746 citations were screened. After titles were screened, 215 abstracts were screened. 110 full texts were reviewed for eligibility, and 17 studies were included in the final synthesis. An additional manual search in April 2025 identified two further eligible studies not retrieved in the original database search. These were included following full-text screening. Figure 1 outlines the screening procedure.

### Study characteristics

Included studies involved a range of premenopausal women (defined in the studies as having regular menstrual periods, younger than 40 years, younger than 45 years, or not having started the menopausal transition), perimenopausal (defined as the onset of menopause symptoms lasting until the cessation of menstruation, women aged 45–55 years, having irregular menstrual periods, cessation of periods permanently for <1 year, aged >40 years, or currently experiencing the menopausal transition), menopausal (defined as at least 1 year without having a menstrual period) and postmenopausal (at least 1 year without having a menstrual period, over 45 years old, over 40 years old, or have completed the menopausal transition). Due to the limited research on menopause, definitions of the stages of menopause are scarce and often inconsistent; therefore, we have adopted the definitions provided by the papers included in this review. Studies were conducted in Europe (*n* = 10), the USA (*n* = 2) and Asia (*n* = 7). The mean age of included participants was 47.56 years (SD = 1.34), indicating that most samples clustered closely around the typical menopausal transition age; however, two studies did not include a mean age or age range. Study characteristics and details are reported in Table 2.

**Table 2.** Studies included in this review.

Author (s) setting	Study design	Participants			Funding	Key outcome measure	Measure of menopause	Relevant finding	Limitations
		Number	Age	Ethnicity					
An et al. (2022). Korea.	Comparative cross-sectional.	45,177 women who underwent regular health check-ups between 2015 and 2018.	Aged between 40 and 65 years. Average age: 47.3 years.	Asian.	No conflict of interest.	Suicidality within the past year measured by self-reported questionnaire.	Premenopause: regular periods. Early menopausal transition: difference of 7+ days in length of menstrual cycles. Late menopause: amenorrhea for 60+ days. Postmenopause: amenorrhea for 12+ months.	3236 women (7.16%) reported suicidal ideation. 1286 (5.73%) cases of suicidal ideation during pre-menopause, 531 (7.57%) during the early menopausal transition, 261 (6.65%) during the late menopausal transition and 1158 (9.8%) during post-menopause.	Self-administered questionnaire instead of clinical diagnosis. Limited generalisability due to a relatively healthy, high socioeconomic status sample of Korean women.
Blackmore et al. (2008). U.K.^a^	Case series.	92 premenopausal and 17 postmenopausal women. 5 postmenopausal women reported experiencing perimenopausal affective episode	Average age: 40 years.	Not reported.	No conflict of interest.	Occurrence of affective episodes in women with a history of postpartum psychosis during perimenopause or postmenopause. Measured with the SCAN (Wing et al., 1990^48^.	Perimenopause: the period starting from onset of symptoms lasting until cessation of menstruation. Postmenopause: not menstruated for at least 1 year including surgical menopause (hysterectomy or ovariectomy).	1/5 cases had suicide attempt at age 44 in perimenopause. Suggests perimenopause as a suicide risk period for women with bipolar disorder.	Relied on self-reported menopausal symptoms without hormonal confirmation. No comparison group of other women with bipolar disorder.
Gojdź et al. (2013). Poland.	Cross-sectional.	221 working female physicians.	Average age: 50.57 years.	Not reported.	No conflict of interest.	Subjective sense of health amongst physicians aged 45–55 years. Measured with The SHP (Sęk, 2000).	Perimenopause: women aged 45–55 years.	8 (3.62%) of subjects reported suicidal thoughts.	Sample limited to Polish female physicians, reducing generalisability.
Hendriks et al. (2024). U.K.	Observational cohort.	1212 women attending a specialist menopause clinic.	Not reported.	Not reported.	No conflict of interest.	PHQ-9 (suicidal ideation; Kroenke et al., 2001) and Greene Climacteric Scale (menopausal symptoms; Greene, 1976).	Patient-defined perimenopause/menopause status at a specialist clinic.	16% reported suicidal ideation on PHQ-9 before starting Hormone Replacement Therapy (HRT; i.e. oestrogen, testosterone, progesterone).	Clinic-based sample, no control group, self-report only.
Hunt et al. (1987). Britain.	Prospective cohort.	4544 women from 21 menopause clinics who were receiving hormone replacement therapy. 11 died by suicide.	Most women were aged between 45 and 54 years.	Not reported.	No conflict of interest.	Mortality among women who had started HRT over a 10-year period. A postal questionnaire was sent out to elicit information on HRT use since entry to the study to enquire about major morbidity.	Natural menopause: cessation of regular menstrual periods. Surgical menopause: hysterectomy with bilateral oophorectomy (removal of uterus and both ovaries). Partial surgical menopause: hysterectomy with at least one ovary intact. Perimenopause: irregular menses. Premenopause: regular menses.	Mortality was significantly lower than expected based on national rates (relative risk 0.58) apart from suicide or suspected suicide (relative risk 2.53), however, 7 of the 11 women who died from this had a psychiatric history before receiving HRT.	Small size. Absence of an internal control group (attempts to recruit such a group were made but failed).
Kim Ji-Su et al. (2021). Korea.^b^	Cross-sectional.	1348 participants. 647 early menopausal women and 647 age-matched general middle-aged group.	From 45 to 55.	Asian.	Supported by the Ministry of Education of the Republic of Korea.	Differences in physical activity, sedentary behaviour and mental health. Measured by the Korean-language version of the Global Physical Activity Questionnaire (Herrmann et al., 2013^49^ ).	Early menopause: women younger than 45 years. Natural menopause: Women aged 45–55 years.	The early menopausal group was 1.5 times more likely to attempt suicidal behaviours than the natural menopause group.	Reliance on self-reported data. Conducted in South Korea, limiting generalisability to other populations.
Kornstein et al. (2010). USA.^a^	Comparative cross-sectional.	950 premenopausal, 380 perimenopausal, 562 postmenopausal.	Aged between 18 and 75.	White: 72.8%, black: 20%, other: 7.2%.	Federal funds from the National Institute of Mental Health.	Presentation and severity of depressive symptoms in women with major depressive disorder in relation to their menopause status. Used data from the STAR*D study (Fava et al., 2003^50^; Rush et al., 2004^51^).	Premenopause: women younger than 40 years (even those who reported having a hysterectomy/being postmenopausal). Perimenopause: women aged 40 who reported not being postmenopausal or having a hysterectomy. Postmenopausal: women aged over 40 who reported being postmenopausal or having a hysterectomy.	Postmenopausal women were more likely to have suicidal ideation than premenopausal women. Perimenopausal women were less likely to have suicidal ideation than postmenopausal women.	Sample restricted to outpatients with nonpsychotic MDD, limiting generalisability. Menopausal status based on age and basic questions, lacking detailed classification. Reliance on self-report and recall, which may be unreliable.
Kułak-Bejda et al. (2023). Poland.	Comparative cross-sectional.	241 women in 2006 and 350 women in 2021.	Average age in 2006: 50.7. Average age in 2021: 51.5.	Not reported.	No conflict of interest.	Severity of depression and menopause symptoms. Used MRS, the BKMI (Kupperman et al., 1953^52^; Schneider et al., 2001^53^), and The Beck Depression Inventory (Beck et al., 1961).	Menopause: over 40 years old. Menopause symptoms measured using BKMO (Kupperman et al., 1953).	Suicidal ideation was reported more in 2021 than in 2006. It increased by 9%.	Low number of women studied. COVID-19 pandemic may have increased the incidence of depression, anxiety and menopausal symptoms.
Murphy et al. (2013). Doha.	Comparative cross-sectional.	41 pre-, peri- and postmenopausal women.	Aged between 40 and 60 years.	Arab.	Qatar National Research Fund, National Priorities Research Programme.	Qualitative data about how Arab women perceive and experience menopause.	Self-reported as either premenopausal, perimenopausal or postmenopausal.	Many of the women believed that suicide during menopause was a recognised issue. Noted that community support from religious involvement served as a protective factor.	Small sample size and limited scope. Findings based on urban women, not reflecting rural experiences.
Nakanishi et al. (2023). Tokyo.	Longitudinal cohort.	2944 mothers.	Average age: 44 years.	Asian.	This work was supported by a Grant-in-Aid for Transformative. Research Areas from the Ministry of Education, Culture, Sports, Science and Technology of Japan.	Suicidal ideation among middle-aged women assessed using the GHQ (Goldberg and Hillier, 1979; Nakagawa and Daibo, 1982)).	Premenopause: not yet started the menopausal transition. Perimenopause: currently experiencing the transition or had started after their baseline survey. Postmenopause: completed the transition.	Suicidal ideation was observed in 7.8% of the participants at baseline and in 9.7% at the follow-up. Follow-up suicidal ideation ranged between 7.9% and 12.9%.	Menopausal stage based on self-report, not validated by hormone levels. Results based on data from Japanese mothers of adolescents, affecting generalisability. No prior information on maternal suicidal ideation before the second-wave survey.
Pinto-Meza et al. (2006). Barcelona^a^	Prospective cohort.	242 women (95 in their menopause)	Average age of the 242 women: 45.29. Average age of the menopausal women: 59.65.	Not reported.	No conflicts of interest.	Severity of depression, including suicidal ideation in women undergoing treatment for major depression with Selective Serotonin Reuptake Inhibitors (SSRIs).	Menopause: not menstruated in past year.	11.58 of the 95 menopausal women (12.2%) had recurrent thoughts of death, suicidal ideation, or a suicide attempt, in comparison to 1.4% of non-menopausal women and 11.5% of men.	Heterogeneous sample with varying diagnoses and depression severity. Self-reported menopausal status without hormone level analysis. Small sample size.
Ryu et al. (2022). Korea.^b^	Cross-sectional.	2232 menopausal women.	Aged between 40 and 65 years.	Asian.	Funded by NRF of Korea grant funded by the Korean government.	Suicidal ideation among menopausal women looking at association between age at menopause and suicidality. Measured using the Korean language version of the PHQ-9.	POI: menopause before the age of 40. Early menopause: menopause between the age or 40 and 45. Menopause: after the age of 45.	Women with POI had significantly higher suicidal ideation (>20%) than those who experienced menopause at a clinically normal age (<10%) or after 45 years of age (>15%).	Cross-sectional design prevents causality assessment. Self-reported suicidal ideation introduces bias. Self-reported age at menopause poses recall bias risk.
Schairer et al. (1996). Sweden.	Prospective cohort.	23,246 women who were prescribed menopausal oestrogens.	The mean age at time of recruitment was 54.5 years.	Not reported.	No conflict of interest.	Cause-specific mortality with a focus on understanding the relationship between HRT and various causes of death in menopausal women.	Menopause: over the age of 35 and being treated for menopause symptoms.	Suicides accounted for 60 of 1472 deaths.	Cross-sectional design prevents causality assessment. Selection bias due to selective prescribing of HRT. Confounding factors not fully quantified.
Sherr et al. (2016). U.K.	Comparative cross-sectional.	170 women.	69 women over the age of 45, 57 women under the age of 45.	25% white, 69.9% black, 5.1% other ethnic minorities.	No conflict of interest.	Mental health outcomes and menopausal symptoms including suicidality among women with HIV. Examined using the PHQ-9. Women above the age of 45 years completed the MENQOL (Hilditch et al., 1996^55^).	Premenopause: younger than 45 years and not reporting any symptoms. Menopause/postmenopause: Older than 45 years and reporting symptoms.	54.1% of the under-45-year-old women and 56.9% of the over-45-year-old women reported suicidal ideation in the previous week.	Cross-sectional design prevents causality assessment.
Son and Lee (2025). Korea.^b^	Cross-sectional.	Women from the KNHANES; *n* ≈ 27,000–28,000 depending on outcome (ideation, plan, attempt).	Women aged 30 years and older (analysis grouped by age: <40, 40–60, ⩾60).	Not reported; nationally representative Korean sample.	Not reported.	Suicidal ideation, plans and attempts (via survey).	Premenopausal: currently menstruating. POI: menopause before age 40. Early menopause: menopause between ages 40 and 45. Menopause: menopause after age 45, considered clinically normal. Menopause was defined as the absence of menstruation for 12 consecutive months.	Higher suicidal ideation and attempts in POI, early and normal menopause vs premenopausal women.	Cross-sectional; self-report; cannot infer causality.
Studd. (2014). London.	Cross-sectional survey.	238 patients at a menopause clinic.	Not reported.	Not reported.	No conflict of interest.	Effectiveness of HRT in treating hormonal-related depression in women. Explored severity of depression (severe, moderate, psychotic episodes and suicide attempts).	Self-reported symptoms of depression related to hormonal events (premenstrual syndrome, post-natal depression and perimenopausal depression).	14% of women had attempted suicide.	None reported.
Usall et al. (2009). Multiple European countries	Cross-sectional.	8794 participants.	Mean age of premenopausal women: 35.62. Mean age of perimenopausal women: 50.42. Mean age of postmenopausal women: 63.67.	Not reported.	Funded by the European Commission.	Prevalence of suicidal ideation over the past year across different stages of the reproductive life cycle in women. Data collected via interview.	Premenopause: still having menstrual periods. Perimenopause: periods stopped temporarily due to the onset of menopause or those who had stopped having periods permanently for <1 year. Postmenopause: periods have stopped permanently for more than 1 year.	Perimenopausal women showed seven times higher prevalence of suicide ideation (7.8%) than premenopause (1.1%) and postmenopause (1%).	Small sample size in some categories. Self-reported menopausal status. Cross-sectional design prevents causal conclusions.
Weiss et al. (2016). USA.^a^	Cross-sectional.	298 women with mood disorders.	Aged between 19 and 90. Average age: 43.1.	82% were Caucasian, 11% of African heritage and 3% were Asian. The remaining 4% were of other varied origins.	No conflict of interest.	Severity of depressive symptoms in relation to various stages of the reproductive life cycle measured by the QIDS-SR (Rush et al., 2003).	Premenopause with regular periods. Premenopause with irregular periods. Pregnant. Perimenopause: irregular periods as menopause approaches. Surgically-induced menopause: uterus and/or ovaries removed. Postmenopause: at least 1 year without a period.	Hormonal status was not a significant predictor of suicide ideation or attempts. 33 % (6/18) of women who had undergone surgical removal of their ovaries or uterus were at high risk for suicide. 12% of all perimenopausal women and 18.5% of all premenopausal women were at risk for suicide.	Did not examine other contributory factors (history of depression, substance use, life stress, genetic/biomarkers). Small sample size.
Won et al. (2016). Korea.^b^	Cross-sectional.	2236 women.	Aged between 19 and 64.	Not reported.	No conflict of interest.	Prevalence of depression. Used data from the first year of the KNHANES VI, performed by the Korean CDC (2013).	Yes/no: ‘have you experienced menopause?’.	6.5% of 19- to 29-year-olds, 4% of 30-49-year-olds and 5.9% of 50- to 64-year-olds reported suicidal thinking in the past year.	Cross-sectional design limits causation.

See Supplemental Table 3 for full details. SHP: subjective health profile; STAR*D: Sequenced Treatment Alternatives to Relieve Depression; MRS: Menopause Rating Scale; BKMI: Blatt–Kupperman Menopausal Index; NRF: National Research Foundation; MENQOL: Menopause Specific Quality of Life questionnaire; QIDS-SR: Quick Inventory of Depressive Symptomatology – Self-Report; CDC: Centers for Disease Control and Prevention; SCAN: Schedule for Assessment in Neuropsychiatry; GHQ: General Health Questionnaire; KNHANES: Korean National Health and Nutrition Examination Survey; BMI: body mass index; POI: primary ovarian insufficiency; PHQ-9: Patient Health Questionnaire-9.

a Psychiatric diagnosis reported.

b BMI data reported.

### Quality assessment

Each study was scored based on specific criteria related to its design. The MMAT used in this review includes two screening questions and additional questions dependent on the study design. These criteria are scored on a nominal scale (yes/no/can’t tell) and allow for the assessment of five main types of studies. Studies were rated as low (0%–40%), medium (40%–60%) or high quality (60%+). The MMAT assessed multiple things to evaluate the quality of studies including the relevance of research questions, appropriateness of methodology, study sample and recruitment, data collection, risk of bias and data analysis and interpretation.

Of the 19 studies, 12 studies scored high in methodological quality (80% or higher), while 7 scored medium (40%–60%). Lower quality ratings were due to small sample sizes (*n* = 2), lack of control groups (n = 1), reliance on retrospective data (*n* = 1), lack of longitudinal data (*n* = 2), lack of detailed statistical analysis (*n* = 1) and inadequate consideration of confounding factors (*n* = 5). See Supplemental Table 2 for further information on MMAT scores and the reasoning behind the assigned scores.

### Suicidality and menopause

The following section reports the primary outcomes of the systematic review: the relationship between suicidality and menopause in those studies that used the term ‘menopause’ as a broad term for the whole transition, without exploring its stages. Some high-quality studies (*n* = 6) reported an increased risk of suicidality during the menopausal transition when compared to pre-menopause. Pinto-Meza et al.^57^ observed a significant increase in suicidality independent of depression symptoms during menopause in a cohort of Spanish women, and Ryu et al.^58^ found similar results in a large-scale cross-sectional study in Korea. Son and Lee,^59^ also using Korean national data, reported that women who had reached menopause had significantly higher odds of suicidal ideation, suicide planning, and suicide attempts compared to premenopausal women, with risk highest among those with premature or early menopause. Won et al.^60^ found the highest levels of suicidal thoughts among women in their 50- to 64-year-old age group, only for those without depression. In the United Kingdom, Sherr et al.^61^ reported 57% of the menopausal women in their study experienced suicidal thoughts in the previous week, and Hunt et al.^62^ found that suicide or suspected suicide rates were higher than expected in a population of women utilising menopause clinics and using Hormone Replacement Therapy (HRT; i.e. oestrogen, progesterone, testosterone), with 11 out of 12 deaths at 10-year follow-up being due to suicide.

Four moderate-quality studies also supported the findings of increased suicide risk during the menopausal transition. Hendriks et al.^63^ found that 16% of women attending a specialist menopause clinic in the United Kingdom reported suicidal ideation or thoughts of self-harm in the 2 weeks prior to their appointment, highlighting the clinical relevance of suicidality in women seeking menopause-related care. Murphy et al.^64^ found in their qualitative study with Arab women, suicidality during menopause was a known issue to them, but it was often attributed to a lack of religion and social support, which they perceived as more characteristic of Western cultures than their own. Kułak-Bejda et al.^65^ observed an increase of 9% in suicidality among menopausal Polish women between 2006 and 2021. However, this research was limited as they did not have a comparison group of non-menopausal women. Studd^66^ reported 14% of women had attempted to die by suicide in their cross-sectional survey of those attending a Premenstrual Syndrome and Menopause Centre in the United Kingdom; however, it is important to note that this centre also treated those with premenstrual syndrome and postnatal depression, thus the sample did not comprise exclusively menopausal women.

### Suicidality and perimenopause

Some high-quality (*n* = 4) and moderate-quality studies (*n* = 3) found that suicidality was more prevalent during the perimenopausal period. An et al.^67^ found that perimenopausal (and post-menopausal) middle-aged Korean women had higher rates of suicidality compared to those who were premenopausal. Specifically, those in perimenopause were 33% more likely to experience suicidality than those who were premenopausal. Nakanishi et al.^32^ reported that perimenopause was associated with the manifestation of suicidal ideation at follow-up, 4–9 years after baseline, even after controlling for prior incidences of suicidality. Similarly, Usall et al.^68^ found a seven-fold increased risk of suicidal ideation among women experiencing perimenopause when compared to pre- and post-menopause, and men of the same age brackets, independent of mood and anxiety disorders; a result they found across six European countries. Gojdź et al.^69^ found that 4% of women from a cohort of Polish female physicians were experiencing suicidal thoughts during perimenopause. Three moderate-quality studies also suggested increased suicide risk during perimenopause. Specifically, Blackmore et al.^70^ found that one woman reviewed for their case series had attempted suicide at age 44 while being actively perimenopausal (i.e. experiencing hot flushes, erratic menstrual periods and poor sleep quality). Studd^66^ also reported that ‘many’ of the 14% of women in his study who had attempted to die by suicide were experiencing perimenopausal depression. Similarly, Hendriks et al.^63^ reported that 16% of women attending a specialist menopause clinic in the United Kingdom, many of whom were actively perimenopausal, experienced suicidal ideation or thoughts of self-harm in the 2 weeks prior to attending their initial appointment. This further supports the observation that suicidality may be a prominent issue during the perimenopausal phase, particularly in clinical populations seeking support for hormone-related symptoms.

### Cultural and social factors and suicidality

The above findings suggesting suicidality is elevated among some women experiencing menopause were consistent across differing cultural contexts, with the effect being observed in studies from Asia,^32,58,60,67,71^ Europe,^57,59,65,68,69^ the United Kingdom^61–63,66,70^ and the Middle East.^64^ This indicates that while cultural nuances such as differing attitudes towards ageing and the availability of support systems exist, the impacts of mental and physical health difficulties on menopause may be universal.

In Sherr et al.’s^61^ study, over half of the menopausal women were found to have experienced suicidal thoughts in the past week, with 69.9% of these women being of Black ethnic origin. Although the authors did not directly examine the influence of ethnicity on suicidality during menopause, the high rate of suicidal ideation underscores the importance of considering how cultural and social factors may contribute.

An et al.^67^ reported that family support – particularly spousal support – was important in protecting against suicidality, and Nakanishi et al.^32^ reported that women who had greater social support at baseline were less likely to experience suicidality at follow-up, 4–9 years after baseline. However, they also reported that menopausal mothers of adolescents with psychological or behavioural problems were more likely to experience suicidality, even when controlling for social support. Kułak-Bejda et al.^65^ noted that increased suicidal ideation during the menopausal transition may be linked to a decrease in social support, as women in their study reported needing more help from spouses and friends but received less as their suicidality increased. Murphy et al.^64^ found that Arab women reported religion playing a role in how they coped with menopause, with it often being a protective factor against suicidality. Women in this study believed Western women to be more vulnerable to suicidal thoughts during menopause due to a lack of religion and thus no sense of community.

Gojdź et al.^69^ reported for perimenopausal women (aged 45–55 years), suicidal thoughts had a statistically significant relationship with a lower self-perception of health. Additionally, a significant relationship was found between suicidal thoughts and physical activity, relationship with physical appearance, and eating habits, as women who exercised more than once a week, were satisfied with how they looked, consumed regular meals, had higher self-perception of health, and reported fewer suicidal thoughts.

However, Son and Lee^59^ found that menopausal women had higher odds of suicidal ideation, planning, and attempts compared to premenopausal women, even after adjusting for socioeconomic and lifestyle factors such as income, employment, BMI, alcohol use, and smoking. This suggests that while social context plays a role, biological and psychological vulnerabilities associated with menopause may transcend cultural boundaries.

### Comorbidities and suicidality

Psychological or physical health conditions impacted on suicidality during menopause.^31,60–62,67,68,70,72^ Blackmore et al.^70^ described a case study of a menopausal woman with bipolar disorder who experienced multiple, severe mental health issues at different stages of her life leading to two suicide attempts, one when she was in perimenopause after a hysterectomy and ovariectomy. An et al.^67^ noted that alcohol use at clinical and sub-clinical levels (⩾10 g of ethanol per day) during menopause was significantly associated with an increase in suicidality, particularly during the later stages of the transition. They also found that, in the later stages, those with comorbid depression had higher suicidality levels. Obesity (BMI = >25) and insomnia were other comorbidities found to increase suicidality in the later stages of menopause.

Hunt et al.^62^ reported that 7 out of the 11 women who died by suicide in their study had a documented history of psychiatric conditions before seeking HRT. Relatedly, Usall et al.^68^ found that comorbid mood and anxiety disorders were associated with an increased risk of suicidality during menopause. This was also supported by Nakanishi et al.^32^ who found that history of depression was a significant predictor of suicidal ideation. Weiss et al.^72^ found that anxiety was the strongest predictor of suicidal thoughts in their cohort of women across the reproductive cycle, including the menopausal transition. Even in studies where no link was found between menopause and suicidality, authors reported that those with psychiatric disorders during this time may be more vulnerable to suicidality.^73^

Sherr et al.^61^ documented that 57% of all menopausal women attending an HIV clinic reported experiencing active suicidal ideation, and the combination of other mental health challenges such as anxiety and depression alongside this was associated with a higher risk of suicidality. Pinto-Meza et al.^57^ found that menopausal women with diagnosed depressive disorders had a poorer response to Selective Serotonin Reuptake Inhibitor (SSRI) treatment and had higher suicidality than non-menopausal women, which suggests that the menopausal transition may interfere with drug treatment for depression, potentially contributing to subsequent suicidality.

Regarding physical comorbidities, Ryu et al.^68^ found that those who had the highest suicidality also had higher rates of chronic diseases such as diabetes, hypertension, and obesity.

### Early menopause and suicidality

Kim Ji-Su et al.^71^ explored early menopause in a cohort of Korean women. They found that women who experienced menopause before the age of 45 were 1.5 times more likely to attempt suicide than the non-early menopausal group. Ryu et al.’s^58^ study echoed these results, finding that those experiencing menopause at an earlier age had a higher incidence of suicidality, even without a diagnosis of a depressive disorder. Son and Lee^59^ further reinforced these findings in a large, nationally representative Korean cohort: women with premature ovarian insufficiency (POI; menopause before age 40) had significantly higher odds of suicidal ideation, suicide planning, and suicide attempts compared to premenopausal women. Early menopause (ages 40–45) was also significantly associated with increased suicidal ideation and suicide planning, although suicide attempts in this group were not statistically significant.

### Studies that did not find increased suicidality

Three studies did not find a significant association between menopausal status and increased suicidality. Schairer et al.^73^ conducted a longitudinal study to examine changes in suicidality over the menopausal transition through mortality analyses; however, their data did not show a significant increase. The authors reported that suicide accounted for most injury-related deaths in their cohort (56%), but when compared to the total number of deaths, suicide was not a dominant cause of mortality (4%). They also found that women using higher doses of hormone replacement therapies were at higher risk for death by suicide compared to those using lower doses and the general population, however, pre-HRT symptom severity was not controlled for, and therefore those using HRT may have been experiencing more detrimental symptoms. Weiss et al.^72^ found that menopausal status did not predict suicidality directly, with perimenopausal women having the lowest risk for suicide. However, they also found that 33% of those experiencing surgically induced menopause in their cohort were classified as high risk for suicidality. Additionally, Kornstein et al.^74^ reported that postmenopausal women had the highest levels of suicidal ideation when compared to both pre-and perimenopausal women, even after controlling for sociodemographic and clinical baseline differences, with perimenopausal women having the lowest levels.

## Discussion

This review provides an important extension to previous syntheses of suicidality in the menopausal transition. While Martin-Key et al.^35^ offered an initial systematic overview, our review uniquely examines cultural and ethnic contexts, the specific risk of early menopause and POI, and the role of comorbid physical health conditions such as metabolic syndrome and high BMI; factors that have not been comprehensively synthesised before. This more nuanced approach helps address the complex interplay of biological, social, and physical health factors in suicidality during menopause. Methodologically, we rigorously followed PRISMA guidelines for study selection and quality assessment and applied the MMAT to appraise the quality of included studies. Given the heterogeneity in study designs, populations, and outcomes, a narrative synthesis^44^ was the most appropriate and transparent approach to integrating diverse evidence while respecting methodological differences. This ensures that our synthesis is systematic and meaningful, despite the lack of pooled effect sizes.

This review aimed to build on previous work by synthesising evidence about suicidality during menopause, with a focus on early menopause, POI, cultural and ethnic factors, and physical health comorbidities, and to highlight implications for integrated care. Key contributions of this review include:

- Systematically highlighting early menopause and POI as unique risk factors for suicidality during menopause, even when depression or anxiety is not present.
- Exploring how cultural and ethnic contexts shape the experience of suicidality during menopause, an area that has received limited attention in previous syntheses.
- Synthesising evidence on comorbid physical health conditions, such as metabolic syndrome and high BMI, which are important but often overlooked contributors to suicidality in this population.

Many included studies reported higher rates or prevalence of suicidality during the menopausal transition, though most were cross-sectional and lacked comparator groups, limiting conclusions about changes in risk.^57,58,60–65,72,73^ Some studies indicated that this effect was independent of prior suicidality,^32^ and mood and anxiety disorders.^68^ Perimenopausal women were particularly vulnerable, experiencing higher rates of suicidality than premenopausal and postmenopausal women.^32,66–69^ except one study, which found post-menopausal women to have the highest level of suicidality.^74^

Three primary factors that could increase suicide risk during menopause emerged from the review: hormonal changes, psychosocial factors, and cultural factors. Hormonal changes were most consistently reported as significant factors that influence suicidality during menopause,^66–69^ as fluctuating levels of oestrogen and progesterone characterise the transition, with oestrogens exerting neuroprotective effects through receptors in the brain.^58^ As oestrogen levels decline, these neuroprotective effects diminish, which can lead to increased vulnerability to depressive symptoms, mood instability, and suicidality.^75^ Two studies noted that hormonal fluctuations influence poor mental health, with no mention of suicidality specifically.^70,71^ Notably, some studies highlighted the perimenopausal stage as particularly vulnerable, which is also the phase characterised by the highest level of hormone fluctuation.^67,68^ This is reflective of the limited research available in this area, which indicates that perimenopause is characterised by decreasing or fluctuating oestrogen and progesterone levels, and suicides in women tend to cluster around these hormonal states,^76,77^ potentially via lower hormone activity reducing serotonergic function.^78^ However, issues of suicidality and potential mediating factors have not been adequately examined in existing research.

Due to the limited direct research on women’s natural hormone levels, to understand the role of hormones in suicidality during menopause, it is important to explore the effects when they are introduced through other means, such as HRT. National Institute for Care and Excellence (NICE) guidance acknowledges that HRT can alleviate low mood and anxiety when these symptoms are associated with menopause, however, it also notes that cognitive behavioural therapy (CBT) may be an option for managing this.^15^ In this review, mixed results regarding HRT were found. Schairer et al.^73^ reported an elevated risk of suicidality among women using more potent oestrogen or combined hormone replacement regimens. On the other hand, Studd^66^ found that over one-third of women in their study answered ‘yes cured’ when asked if HRT helped their depression, and over half reported a significant improvement; a factor that can indirectly lower suicidality. When looking at the wider scope of literature regarding HRT and suicidality, it is limited. A recent review by Herson and Kulkarni^79^ discusses that treatments with HRT are an obvious option for depression during menopause, citing several small randomised controlled trials that demonstrate its effectiveness.^80–82^. However, in their systematic review, Stacy et al.^77^ discussed a large retrospective longitudinal study in a veteran population that found more than a two-fold increase in suicide-related deaths among HRT users, but the relationship weakened after accounting for comorbid psychiatric conditions and medication usage.^83^ Further, larger clinical trials are required as direct research on the effects of HRT on suicidality is lacking.

Early menopause (i.e. perimenopause starting between the ages of 40 and 44)^15^ was identified as a particularly severe risk factor for suicidality during menopause as Kim Ji-Su et al.^71^ found those who reported experiencing menopause before the age of 45 were one and a half times more likely to experience suicidal ideations. Research exploring early menopause primarily focuses on general mental health outcomes such as depression and anxiety rather than specifically addressing suicidality, however, it suggests that early menopause is associated with impaired mental health.^84^ Additionally, Kim Ji-Su et al. found their participants to have higher perceived stress, also known to be a risk factor for suicidality at other points of the female lifespan.^85^

Furthermore, Ryu et al. found that age at menopause was significantly associated with a prevalence of suicidal ideation, with their POI (i.e. perimenopause starting before 40 years)^15^ cohort having a four-fold higher risk than their regular menopause group; a finding also reinforced by Son and Lee,^59^ who reported that women with POI had significantly greater odds of suicidal ideation (OR = 1.53), planning (OR = 1.92), and attempts (OR = 2.01) compared to premenopausal women, even after adjusting for depression, BMI and alcohol use.

Ryu et al.^58^ also found that those with POI had higher rates of chronic illness such as diabetes, obesity, and hypertension, which is reflective of other research in the area.^86,87^ Women with chronic illness are at a higher risk for suicidality, as chronic health conditions can exacerbate psychological distress and contribute to increased vulnerability.^88^ Given previous research in the area suggests that POI in general is associated with impaired mental health,^89^ screening for POI in healthcare settings is crucial, as these individuals may be at a higher risk for developing suicidality due to the compounding effects of their condition and the menopausal transition. Additionally, a cross-sectional study found that one-third of women with POI who also suffer from anxiety or depression are underdiagnosed,^90^ further highlighting the need for more comprehensive mental health screening for this population.

Physical symptoms commonly experienced during menopause, such as hot flashes, sleep disturbances, and joint pain, are also strongly linked to poor mental health outcomes in menopausal women without POI.^91^ Weiss et al.^72^ found that these physical health challenges, when combined with anxiety, significantly elevated the severity of depressive symptoms in menopausal women, heightening the likelihood of suicidal ideation. Therefore, addressing both physical and mental health aspects of menopause is essential to mitigate the risk of suicidality during this time. However, it is notable that most studies did not report BMI data – only four studies^59,60,71,75^ provided this information – limiting our understanding of how metabolic health may intersect with suicidality during this transitional phase. Given the significant metabolic changes that occur during menopause^92^ and their relevance for mental health, including increased suicidality,^93,94^ highlights the need for future research and clinical practice to address the interplay of physical and mental health factors in menopausal suicidality.

The review also found that psychosocial factors, including pre-existing psychological conditions and social support, significantly influenced suicidality during the menopausal transition.^61,64^ Women with a history of depressive or anxiety disorders were at a higher risk of suicidality during menopause, with some studies suggesting that the addition of hormonal changes at a time when someone is also experiencing psychological difficulties – which could be attributed to, or separate from hormonal activity – could exacerbate these symptoms and thus lead to suicidal thoughts.^68,70^ Previous research indicates that psychiatric diagnoses of general anxiety disorder^95^ and major depressive disorder^96^ may be associated with reduced resilience to deal with life stressors. This loss of resilience may further heighten the risk of suicidality during menopause, as women face both the psychological burden of hormonal fluctuations and reduced ability to cope with additional stressors as they normally would. Contrastingly, two studies found suicidality to be higher during menopause, independent of prior suicidal ideation^32^ and comorbid mood disorders.^68^

Heightened alcohol use during menopause was associated with an increase in suicidality,^67^ reflecting patterns seen at other times in the lifespan, specifically with women.^97^ There is evidence that oestrogens and progesterone can influence alcohol intake^98^ with animal studies showing that higher levels of oestrogen increase neural excitability, synaptic transmission, and the metabolism rate of ethanol.^99^ This may be why women find alcohol more rewarding during the follicular phase of the menstrual cycle, when oestrogen levels are highest.^100^ It is also known that oestrogen levels can be up to 30% higher during perimenopause when compared to premenopause^101^ before they become erratic and eventually settle into low levels,^102^ potentially driving similar rewarding patterns of increased alcohol use. Times of hormonal fluctuation such as the postpartum period have also been linked to increased alcohol use,^103^ and the unpredictable hormonal shifts during perimenopause may similarly lead to this outcome. Given these risks, it is critical that alcohol screening and support services are integrated into routine menopause care for women to reduce the risk of suicidality.

Social support, particularly from family and spouses, was found to be a protective factor against suicidality in some studies, and social isolation and lack of community or support increased suicide risk.^32,61,65^ The Women’s Health Strategy for England^104^ has highlighted the significance of mental and social well-being in the health outcomes of menopausal women and has recognised the current gaps in this type of care. To help bridge this gap, The Menopause Café initiative, introduced in 2017, offers a community-based approach that addresses this need for social connection among menopausal women, providing a safe, non-judgemental space for discussing menopause-related experiences and challenges.^105^ Notably, Menopause Cafés not only welcome those experiencing menopause but also partners, family members, and friends as per their official website.^106^ This inclusivity strengthens the support network by enabling loved ones to better understand menopause and actively participate in supportive conversations. Such supportive environments can foster peer support, reduce social isolation, and promote emotional well-being, which may mitigate suicide risk.^77^

Cultural influences particularly societal attitudes towards menopause also played a role in increasing the risk of suicidality.^64^ These articles indicated that in cultures where menopause is viewed negatively or stigmatised, women reported higher levels of suicidality. Contrarily, in cultures where menopause is viewed positively and seen as a natural part of life, women experienced lower rates of suicidal ideation.^64^ This suggests that cultural perceptions are important in how women experience and cope with the symptoms of menopause, including suicidality. However, while it’s important to acknowledge the challenges faced during menopause, framing menopause predominantly in terms of disability – as suggested by recent Equality and Human Rights Commission^107^ guidance – may contribute to the stigmatism and ageism already faced by this population.^108^

The mental health of menopausal women can be particularly at risk when combined with the demands of parenting, especially for those raising children who may have developmental, psychological, or behavioural issues. While only one study in this review directly links such parenting challenges with suicidality during menopause,^32^ broader research consistently demonstrates that mothers in these roles generally face elevated mental health difficulties such as stress, anxiety, depression, and suicidality.^109,110^ Research also highlights the significance of building resilience when raising children with conditions such as these,^111^ a characteristic that becomes challenged in menopause.^112^ This loss of resilience, compounded by hormonal shifts and physical changes during this time could make the cumulative stress feel overwhelming, potentially heightening the risk of suicidality. Supporting mothers of children with complex needs is crucial, particularly during menopause, and dedicated services are essential to provide this population with assistance in navigating the combined pressures of menopausal changes and challenging parenting.

### Strengths and limitations

This systematic review is the first to comprehensively examine suicidality during the menopausal transition from multiple perspectives, including hormonal, psychosocial, and cultural. The review methodology was consistent with the most up-to-date standards (PRISMA guidelines) for study selection, data extraction and quality assessment. However, the review has several limitations. First, the number of overall included studies is limited, which is reflective of the lack of research in this area. There are also a small number of studies that focus specifically on the perimenopausal stage, which limits the ability to draw any categorical conclusions about this critical period of the menopausal transition. There were no studies that explored self-harm or non-suicidal self-injury, which is a significant gap as these behaviours are important mental health concerns that often co-occur with suicidality but serve different psychological functions.^113^ Failing to consider these behaviours limits the scope of understanding the full range of challenges menopausal women may face, as many turn to self-harm to cope with symptoms.^63^ Although studies such as Clements et al.^114^ provide important insight into self-harm and suicide among midlife women aged 40–59, the lack of menopausal staging meant the study did not meet inclusion criteria. This highlights the need for future research to clearly define menopausal status when exploring self-harm behaviours.

Another limitation is the reliance on self-reported data, which may introduce bias and impact the reliability of the findings. Future research may utilise a menopause symptom tool such as the Greene Climacteric Scale.^115^ Studies were also only included if they were published in the English language, or where an English translation was available, so further research on cultural differences may exist but were not included in this review. This may have limited the review’s ability to capture the full scope of the relationship between menopause and suicidality, particularly in contexts where cultural attitudes towards menopause may differ. Additionally, the small number of longitudinal and RCT studies further limits exploring the causal association between suicidality and menopause.

Another limitation would be the inconsistency in distinguishing menopausal stages across the included studies. This lack of standardisation makes direct comparisons across studies challenging and may contribute to variation in the findings. Futhermore, the focus on published studies may introduce publication bias, as studies that have significant results are more likely to be published than those with non-significant findings.^116,117^

Additionally, most included studies were observational, with the majority being cross-sectional and lacking comparator groups (see Table 2). As such, this review cannot draw conclusions about changes in suicidality risk over time or causal associations. While several studies reported increased suicidality during the menopausal transition, these findings must be interpreted cautiously. Moreover, the inclusion of qualitative and quantitative studies necessitated a narrative synthesis rather than a statistical meta-analysis, which limits generalisability. Finally, the use of MMAT scores to label studies as high or medium quality does not override fundamental limitations inherent to certain study designs, particularly cross-sectional research. Although several cross-sectional studies were rated as ‘high quality’ using the MMAT, we acknowledge that cross-sectional designs are limited in their ability to establish causality or track change over time. However, consistent with MMAT guidance, these studies were assessed based on the rigour with which they applied their design, rather than being penalised for the design type itself. This is aligned with existing literature supporting fair and context-specific quality appraisal of cross-sectional research.^38,42^

### Implications for clinical practice

While definitive conclusions about causality cannot be drawn, the findings suggest that suicidality during menopause warrants clinical attention. The current review highlights the importance of incorporating psychological screening and support into the care pathway for those going through menopause. Healthcare providers should be aware of the heightened risk of suicidality during the menopausal transition and actively assess for mental health symptoms such as depression, anxiety, and suicidal ideation as part of routine menopause care. Regular screening tools such as the Patient Health Questionnaire-9^118^ for depression could be beneficial in identifying at-risk individuals, as it includes a specific question that focuses on thoughts of suicide or self-harm, and is reasonably effective in detecting suicidality.^119^ Alternatively, the Perimenopausal Depression Scale^120^ could be utilised as it is specific to depression experienced during menopause and includes a question regarding suicidality.

This review indicates the need for a holistic approach to menopause care that includes considering psychosocial factors. Social support networks, including counselling services and peer support groups, should be made available to those suffering from mental ill-health during the menopausal transition phases, especially those with a history of mental health issues or those experiencing social isolation.^64,80^ Screening for increased alcohol use, often noted during menopause, with access to relevant support services could further mitigate suicide risk. Menopause Cafés – a supportive and safe environment for women to openly discuss their experiences with menopause – were first introduced in the United Kingdom in 2017 and have been instrumental in improving mental well-being during menopause.^105^ There have been more than 250 Menopause Cafés that have taken place in the United Kingdom and Canada since 2017, and they have been described as the most successful way to facilitate social cohesion and the most effective method of reducing loneliness and stigmatisation experienced by menopausal women.^105^ However, research is needed to confirm these findings. If supported, expanding the availability of Menopause Cafés could provide a crucial and accessible resource for women, possibly alleviating mental health symptoms experienced during the menopausal transition.

Increased access to services that include CBT or mindfulness-based stress reduction may be helpful, as interventions utilising these techniques have been shown to alleviate depressive symptoms and improve overall well-being in menopausal women^121,122^ and NICE^15^ recommends CBT as a treatment option for depressive symptoms during menopause. Additionally, educational interventions that provide information to women and their families about the potential psychological challenges of menopause can help reduce stigma and encourage early intervention, as research shows many women have limited knowledge about menopause throughout their lives,^123^ however, it is essential that these educational efforts present menopause as a natural life stage rather than framing it as a disability or medicalised condition, which could inadvertently reinforce negative stereotypes.^108^ Targeted support for women with early menopause or POI, who face a significantly higher suicidality risk is also essential for addressing their mental and physical health needs.

Cultural competence in clinical practice is crucial for menopause care, as societal attitudes towards the transition can significantly impact women’s mental health. Healthcare providers should be sensitive to cultural differences and tailor their treatment plans accordingly, offering culturally appropriate support and resources. Research shows that 45% of Black and minority menopausal women experience delays in diagnosis and therefore do not get sufficient and timely care.^31^ People from different cultural and ethnic backgrounds may also have different beliefs and attitudes towards menopause, which can have an impact on their ability to seek help, and their ability to adhere to psychological interventions.^124,125^

Lastly, women who manage the combined challenges of menopause and parenting children with complex needs are at heightened mental health risk, suggesting a need for specialised mental health support for this population. In the United Kingdom, some initiatives provide targeted support for non-menopausal mothers in this position, although resources remain limited and are often challenging to access.^126^ Currently, there are no dedicated support services in the United Kingdom specifically designed to address the unique challenges faced by menopausal women in this position, leaving a critical gap in mental health support for this vulnerable population.

## Conclusion

Overall, the findings suggest that healthcare systems should prioritise the development of integrated care models that consider the multifaceted needs of those experiencing the menopausal transition. This includes medical treatment, mental health support, and social support services to ensure more comprehensive care. The integration of mental health services and screening into routine menopause care could help identify and support women at risk of suicidality, equipping them with knowledge and skills to combat psychological stressors themselve. Enhanced training for clinicians in recognising menopause-induced mental health issues is also imperative, as many medical programmes do not include detailed menopause-focused modules within their curriculum.^127^ Offering continued education and specialised training on menopause to practising clinicians, specifically related to mental health issues and risk of suicide during menopause, would ensure that they are better equipped to provide support to women experiencing menopause transitions.

## Supplemental Material

sj-docx-1-whe-10.1177_17455057251360517 – Supplemental material for Menopause and suicide: A systematic review Supplemental material, sj-docx-1-whe-10.1177_17455057251360517 for Menopause and suicide: A systematic review by Olivia Hendriks, Jason C. McIntyre, Abigail K. Rose, Laura Sambrook, Daniel Reisel, Clair Crockett, Louise Newson and Pooja Saini in Women’s Health

sj-docx-2-whe-10.1177_17455057251360517 – Supplemental material for Menopause and suicide: A systematic review Supplemental material, sj-docx-2-whe-10.1177_17455057251360517 for Menopause and suicide: A systematic review by Olivia Hendriks, Jason C. McIntyre, Abigail K. Rose, Laura Sambrook, Daniel Reisel, Clair Crockett, Louise Newson and Pooja Saini in Women’s Health
