# Supplementary material for: Menopause and suicide: A systematic review
Source: Womens Health (Lond). 2025 Oct 9;21:17455057251360517. doi: 10.1177/17455057251360517 (PMC12515336; doi:10.1177/17455057251360517)
Supplement: sj-docx-2-whe-10.1177_17455057251360517 – Supplemental material for Menopause and suicide: A systematic review [file sj-docx-2-whe-10.1177_17455057251360517.docx]

**Supplementary Materials**

**Supplementary Table 1.** Similarities and Differences Between Included Studies.

|  | **Population Characteristics** | **Definitions** | **Type of Study** |
| --- | --- | --- | --- |
| **Similarities** | Most studies focused on women in the menopausal age range (An et al., 2022; Blackmore et al., 2008; Gojdz et al., 2013; Hunt et al., 1987; Kim Ji-Su et al., 2021; Kułak-Bejda et al., 2023; Murphy et al., 2013; Nakanishi et al., 2023; Pinto-Meza et al., 2006; Ryu et al., 2022; Schairer et al., 1997; Studd, 2014).  Most studies did not consider socioeconomic and cultural factors and their effect on menopause (Blackmore et al., 2008; Hunt et al., 1987; Kim Ji-Su et al., 2021; Kornstein et al., 2010; Pinto-Meza et al., 2006; Ryu et al., 2022; Schairer et al., 1997; Sherr et al., 2016; Studd, 2014; Usall et al., 2009; Weiss et al., 2016; Won et al., 2016).  Most of the studies did explore pre-existing health conditions and their effect on menopause-related health outcomes, including alcohol use (An et al., 2022), psychiatric conditions (Blackmore et al., 2008; Hunt et al., 1987; Nakanishi et al., 2023; Schairer et al., 1997; Usall et al., 2009; Weiss et al., 2016; Won et al., 2016), and physical health conditions (Ryu et al., 2022; Sherr et al., 2016). | All studies gave clear definitions of menopause as being the cessation of menstrual periods after one year (An et al., 2022; Blackmore et al., 2008; Gojdz et al., 2013; Hunt et al., 1987; Kim Ji-Su et al., 2021; Kornstein et al., 2010; Kułak-Bejda et al., 2023; Murphy et al., 2013; Nakanishi et al., 2023; Pinto-Meza et al., 2006; Ryu et al., 2022; Schairer et al., 1997; Sherr et al., 2016; Studd, 2014; Usall et al., 2009; Weiss et al., 2016; Won et al., 2016).  All studies defined suicidality as either thoughts, attempts or completed suicide, with none including self-harm/suicidal self-injury (An et al., 2022; Blackmore et al., 2008; Gojdz et al., 2013; Hunt et al., 1987; Kim Ji-Su et al., 2021; Kornstein et al., 2010; Kułak-Bejda et al., 2023; Murphy et al., 2013; Nakanishi et al., 2023; Pinto-Meza et al., 2006; Ryu et al., 2022; Schairer et al., 1997; Sherr et al., 2016; Studd, 2014; Usall et al., 2009; Weiss et al., 2016; Won et al., 2016).  Most studies separated the menopausal transition into two or three stages i.e. premenopause, perimenopause and/or postmenopause (An et al., 2022; Blackmore et al., 2008; Hunt et al., 1987; Kornstein et al., 2010; Murphy et al., 2013; Nakanishi et al., 2023; Sherr et al., 2016; Usall et al., 2009; Weiss et al., 2016). | Most studies were cross-sectional in design (An et al., 2022; Gojdz et al., 2013; Kim Ji-Su et al., 2021; Kornstein et al., 2010; Kułak-Bejda et al., 2023; Murphy et al., 2013; Ryu et al., 2022; Sherr er al., 2016; Studd, 2014; Usall et al., 2009; Weiss et al., 2016; Won et al., 2016).  Most studies used self-report methods for gathering data on menopausal symptoms and suicidality (An et al., 2010; Blackmore et al., 2008; Gojdz et al., 2013; Hunt et al., 1987; Kim Ji-Su et al., 2021; Kornstein et al., 2010; Kułak-Bejda et al., 2023; Murphy et al., 2013; Nakanishi dt al., 2023; Pinto-Meza et al., 2006; Ryu et al., 2022; Sherr et al., 2016; Studd, 2014; Usall et al., 2009; Weiss et al., 2016; Won et al., 2016).  Most studies were observational (An et al., 2022; Blackmore et al., 2008; Gojdz et al., 2013; Kim Ji-Su et al., 2021; Kornstein et al., 2010; Kułak-Bejda et al., 2023; Murphy et al., 2013; Nakanishi et al., 2023; Pinto-Meza et al., 2006; Ryu et al., 2022; Sherr et al., 2016; Usall et al., 2009; Weiss et al., 2016; Won et al., 2016). |
| **Differences** | Age range varied, exploring all ages from 18 onwards (Kornstein et al., 2010; Sherr et al., 2016; Usall et al., 2009; Weiss et al., 2016; Won et al., 2016).  Some studies did consider the impact of socioeconomic and cultural factors on menopause-related health outcomes, including social support (An et al., 2022; Kułak-Bejda et al., 2023; Murphy et al., 2013; Nakanishi et al., 2023) and self-perception (Gojdz et al., 2013).  Some of the studies did not look at comorbidities and their effect on menopause (Gojdz et al., 2013; Kim Ji-Su et al., 2021; Kornstein et al., 2010; Kułak-Bejda et al., 2023; Murphy et al., 2103; Pinto-Meza et al., 2006; Studd, 2014). | Some studies only included one menopausal stage (Gojdz et al., 2013; Kułak-Bejda et al., 2023; Pinto-Meza et al., 2006; Schairer et al., 1997; Studd, 2014; Won et al., 2016).  Few studies mentioned surgically induced menopause (Blackmore et al., 2008; Hunt et al., 1987; Kornstein et al., 2010; Weiss et al., 2016).  Two studies explored the time of life menopause started at i.e. early or natural (Kim Ji-Su et al., 2021; Ryu et al., 2022). | One was a case series (Blackmore et al., 2008), three were prospective cohort (Hunt et al., 1987; Pinto-Meza et al., 2006; Schairer et al., 1996) and one was a longitudinal cohort (Nakanishi et al., 2023).  Two studies were post-mortem analyses (Schairer et al. 1997; Hunt et al., 1987).  Two studies looked at the effect of HRT on suicidality (Hunt et al. 1987; Schairer et al., 1997). |

**Supplementary Table 2.** Mixed Methods Appraisal Tool Results for Included Studies.

| **Study ID** | **Study design** | **MMAT criteria** | **MMAT Score (%)** | **Reason for not scoring maximum** |
| --- | --- | --- | --- | --- |
| An et al. (2022) | Comparative cross-sectional | 4/5 | 80% | Limited consideration of confounding factors (No detailed control for potential confounders influencing depressive symptoms and suicidality). |
| Blackmore et al. (2008) | Case series | 3/5 | 60% | Small sample size (5 cases), lack of control group, reliance on retrospective data collection. |
| Gojdz et al. (2013) | Cross-sectional | 4/5 | 80% | Limited consideration of confounding factors (No detailed control for sociodemographic variables and lifestyle-related variable). |
| Hunt et al. (1987) | Prospective cohort | 3/5 | 60% | Limited consideration of confounding factors (Did not account for all potential confounders such as socioeconomic status and lifestyle factors in the analysis), does not provide detailed statistical analyses of the data. |
| Kim et al. (2021) | Cross-sectional | 3/5 | 60% | Limited consideration of confounding factors (No detailed control for potential confounders influencing depressive symptoms and suicidality), limited generalisability. |
| Kornstein et al. (2010) | Comparative cross-sectional | 4/5 | 80% | Limited consideration of confounding factors (Relied on self-reported data, which can introduce bias, and did not fully control for all potential confounding variables such as hormonal therapy use, comorbid mental health conditions, and socio-economic status). |
| Kułak-Bejda et al. (2023) | Comparative cross-sectional | 3/5 | 60% | Limited consideration of confounding factors (No multivariate analysis), reliance on self-reported data. |
| Murphy et al. (2013) | Comparative cross-sectional | 3/5 | 60% | Small sample size (41 participants), potential bias in focus group composition, lack of control for confounding factors (did not control for variables such as educational level, marital status, and cultural differences that might affect perceptions of menopause). |
| Nakanishi et al. (2023) | Longitudinal cohort | 4/5 | 80% | Limited consideration of confounding factors (No detailed control for hormonal and lifestyle variables that might influence outcomes). |
| Pinto-Meza et al. (2006) | Prospective cohort | 4/5 | 80% | Limited control for confounding factors (Did not control for all sociodemographic variables and health behaviours that could influence the outcomes such as smoking, alcohol consumption, and mental health history). |
| Ryu et al. (2022) | Cross-sectional | 4/5 | 80% | Limited control for confounding factors (No detailed adjustment for potential confounders such as lifestyle factors and mental health history). |
| Schairer et al. (1996) | Prospective cohort | 4/5 | 80% | Limited control for confounding factors (Did not fully account for variations in healthcare access and genetic predispositions). |
| Sherr et al. (2016) | Comparative cross-sectional | 4/5 | 80% | Limited control for confounding factors (No detailed control for socioeconomic status and other potential confounders influencing mental health outcomes such as access to support services). |
| Studd (2014) | Cross-sectional survey | 3/5 | 60% | Potential selection bias (Participants self-selected to join the study, possibly leading to non-representative sample), limited control for confounding factors such as socioeconomic status and pre-existing health conditions. |
| Usall et al. (2009) | Cross-sectional | 4/5 | 80% | Limited control for confounding factors (Relied on self-reported menopausal status, no hormonal validation, and limited control for other psychosocial factors). |
| Weiss et al. (2016) | Cross-sectional | 4/5 | 80% | Retrospective data collection issues (Possible recall bias), limited control for confounding factors such as history of depression and lifestyle factors. |
| Won et al. (2016) | Cross-sectional | 4/5 | 80% | Potential recall bias (Relied on self-reported data from past year), limited control for unmeasured confounders such as stress levels and environmental factors. |
